# Spontaneous rectus sheath hematoma associated with apixaban in an elderly gentleman with chronic obstructive airway disease – a case report

**DOI:** 10.1186/s12959-022-00420-z

**Published:** 2022-10-03

**Authors:** Cheuk-Lik Wong, Clarence Hao-Yu So

**Affiliations:** Department of Medicine and Geriatrics, Caritas Medical Centre, 111 Wing Hong Street, Shamshuipo, Kowloon, Hong Kong SAR

**Keywords:** Rectus sheath hematoma, Apixaban, Direct-acting oral anticoagulant, Chronic obstructive airway disease

## Abstract

**Background:**

Rectus sheath hematoma (RSH) is a relatively uncommon cause of acute abdominal pain and can be mistaken as other surgical causes of acute abdomen. A diagnosis requires high index of suspicion especially in susceptible patients, for example, in patients on anticoagulation. While anticoagulation is the commonest risk factor for RSH, direct-acting oral anticoagulants have only been very recently implicated as a potential cause with fewer than ten cases reported in the literature.

Case presentation.

An 82-year-old Chinese man with chronic obstructive airway disease, ischemic heart disease, heart failure and atrial fibrillation on apixaban presenting with acute onset of lower abdominal pain. Physical examination showed peritoneal signs with tenderness and guarding over the lower quadrants with hypotension. Computed tomography (CT) of the abdomen confirmed a large rectus sheath hematoma (RSH) without active extravasation. He was given fluid resuscitation and was managed successfully with supportive treatment and cessation of apixaban. A follow-up CT two months later showed resolving hematoma and aspirin was resumed primarily for ischemic heart disease. The patient tolerated anti-platelet therapy without recurrence of RSH. The risk factors, treatment options, prognosis and issue related to anticoagulation resumption after an episode of RSH are discussed. Reported cases of RSH associated with direct-acting oral anticoagulants are reviewed.

**Conclusions:**

Direct-acting oral anticoagulant-associated rectus sheath hematoma is rare. With increasing use of direct-acting oral anticoagulants in multiple clinical settings, clinicians should remain vigilant of this potentially life-threatening bleeding complication when a patient presents with acute abdominal pain. Conservative treatment with cessation of anti-coagulant and supportive transfusion remains the mainstay of treatment.

## Background

Rectus sheath hematoma (RSH) is a relatively uncommon cause of acute abdominal pain and accounts for approximately 2% of patients presenting with such symptom to the emergency department [1]. Without appropriate investigations such as computed tomography of the abdomen, it is often mistaken as other surgical causes of acute abdomen. A wrong diagnosis may result in inadvertent laparotomy and potential mortality while the majority of cases of RSH can be successfully treated with conservative treatment. As such, a diagnosis requires high index of suspicion especially in susceptible patients, for example, in patients on anticoagulation and in patients with chronic obstructive airway disease especially after coughing bouts [2]. While anticoagulation is the commonest risk factor for RSH, direct-acting oral anticoagulants (DOAC) have been very recently implicated as a potential cause. We herein report a case of an elderly gentleman with chronic obstructive airway disease sustaining spontaneous RSH while receiving apixaban as stroke prophylaxis for atrial fibrillation. The management and prognosis of RSH are discussed and reported cases of DOAC-associated RSH are reviewed. With more widespread use of DOAC in multiple clinical settings, clinicians should remain vigilant of this potentially life-threatening hemorrhagic complication when a patient on DOAC presents with acute abdominal pain.

## Case presentation

An eighty-two-year-old gentleman was admitted to hospital for acute onset of lower abdominal pain for a day. His past health included ischemic heart disease, atrial fibrillation, heart failure and chronic obstructive airway disease (COAD), and was on apixaban for stroke prophylaxis. He was hospitalized a week ago for infective exacerbation of COAD due to Hemophilus influenzae pneumonia which was treated with a 7-day course of amoxicillin-clavulanic acid. He reported repeated coughing bouts the day before admission. On the other hand, there was no history of trauma, abdominal surgery or percutaneous puncture over the abdominal region.Symptoms of gastrointestinal bleeding were absent.

On examination, the patient appeared to be apprehensive and dehydrated. His blood pressure was 99/63 mmHg with a pulse of 67 beats per minute. Abdomen was mildly distended with exquisite tenderness and guarding over the left lower quadrant. Grey Turner’s and Cullen signs were absent. Bowel sound and rectal examination were normal. Laboratory studies showed a hemoglobin of 9.5 g/dL (reference range [RR]: 13.4 to 17.1 g/dL), platelet count 150 × 10^9^/L (RR: 145–370 × 10^9^/L, prothrombin time (PT) of 19.9 s (RR: 9.9–12.3 s; taken at around 12 h after last dose of apixaban), activated partial thromboplastic time (aPTT) of 33.6 s (RR: 28.2–38.2 s), creatinine 108umol/L (RR: 65-109umol/L) and eGFR 55 ml/min/1.73m^2^. His hemoglobin level was 13.3 g/dL one week ago.

**Fig. 1:**
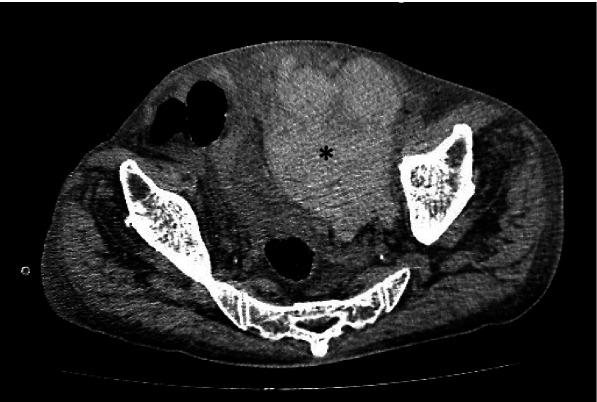
Plain CT abdomen: A hyperdense non-enhancing mass lesion (*) is noted involving the left anterior abdominal wall at the rectus sheath, extending into the prevesical space, measuring at least 10.2 cm × 8.5 cm × 17.3 cm (AP x W x CC) representing a hematoma

**Fig. 2:**
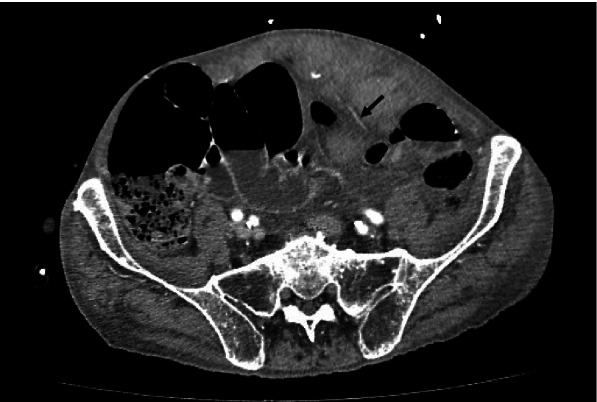
Contrast CT abdomen: Prominent serpiginous enhancing structures within (arrow) representing prominent tortuous vessels. Contrast extravasation was absent

An urgent CT abdomen and pelvis with contrast showed a hyperdense 10 × 8.5 × 17.3 cm (APxWxCC) non-enhancing mass lesion involving the left anterior abdominal wall at the rectus sheath (Fig. 1). It extended into the prevesical space, representing a large rectus sheath haematoma (RSH). Active extravasation was not noted. There was also evidence of contrast reflux from right atrium into IVC and hepatic veins due to underlying heart failure. Prominent serpiginous enhancing structures within the hematoma, representing prominent tortuous vessels probably due to right heart failure, was also noted (Fig. 2).

The patient was treated with fluid resuscitation and analgesics, and apixaban was withheld immediately. His blood pressure improved and the RSH was managed expectantly in the absence of evidence of ongoing bleeding. Reversal with activated prothrombin complex concentrate or blood transfusion was not required. His hemoglobin level nadired at 9.5 g/dL two days later and improved gradually afterwards. A repeat PT two days later became normal at 12.3 s. His symptoms improved significantly over the next few weeks. An echocardiogram later showed dilated right ventricle with a diameter of 3 cm with severe tricuspid regurgitation and elevated right ventricular systolic pressure of 69 mmHg, which was compatible with a diagnosis of cor pulmonale.

The patient’s clinical course was complicated with an episode of methicillin-resistant staphylococcocus aureus pneumonia for which he received a course of vancomycin. He received an extended course of rehabilitation and was discharged about a month later. A follow-up CT scan 2 months later showed a resolving hematoma of 4.5 cm × 4.5 cm × 5.3 cm in size. Aspirin 81 mg daily was resumed at that juncture in lieu of anticoagulation primarily for ischemic heart disease after balancing the risk and benefit of anticoagulation and shared decision with the patient and his family. The patient did not experience any further hemorrhagic complications for the next year.

## Discussion

Spontaneous rectus sheath hematoma (RSH) is an uncommon clinical condition. It accounts for approximately 2% of patients presenting with acute abdominal pain and its incidence has been estimated to be 1.2–1.5 cases per year in patients referred for radiological evaluation of acute abdominal pain [1]. It often results from the rupture of branches of epigastric vessels at its insertion into the rectus abdominis muscle, and less commonly a direct tear of the rectus muscles. Anatomically, the inferior epigastric arteries (IEA) are more prone to injury, and given the absence of posterior rectus sheath below the arcuate line and the presence of aponeurosis in front of the rectus muscles all the way from the umbilicus to pubis, hematomas due to IEA rupture tend to be more extensive and can extend beyond midline, inferiorly into the prevesical space and posteriorly into the peritoneum [2–6].

Anticoagulation is the commonest risk factor for RSH. Antiplatelet agents such as aspirin and clopidogrel have also been implicated. In a large series of 126 cases with RSH, almost 70% of patients were on anticoagulation therapy (primarily warfarin and heparin) prior to the development of RSH [3]. In another retrospective cohort of 115 patients with RSH, 77.4% were on anticoagulants and 30% were on antiplatelet therapy [7]. Other risk factors include intense coughing which could result from COAD or its exacerbation, pregnancy and various medical conditions like hypertension, liver cirrhosis and chronic kidney disease [7]. The abnormally dilated epigastric venules related to cor pulmonale and right heart failure observed on CT imaging likely exacerbated the severity of hemorrhage in the present case. In addition, atheromatous changes in epigastric venules have been identified as a contributing factor for spontaneous RSH in elderly [5]. Although vascular malformation has been reported as a rare cause for RSH and was not completely excluded in our patient by angiography [8], we believe that intense coughing bouts due to COAD exacerbation in an elderly patient in the presence of apixaban were adequate trigger for the incident RSH. In addition, invasive angiography, which is usually reserved for arterial embolization in hemodynamically compromised patients, was not considered in our patient whose RSH occurred singly and was managed successfully with a conservative approach.

More recently, direct-acting oral anticoagulants (DOAC) have been identified as an emerging etiology of spontaneous RSH in the literature. As the time of writing, only 6 case reports of DOAC-associated RSH, with three associated with apixaban and three associated with rivaroxaban [5, 9–13]. The majority of patients were female and all of them were older than 65 years of age. Drug interaction and reduced renal function may be contributing factors to the development of RSH in some of the cases (Table 1) [5, 9–13]. All patients were managed conservatively with one mortality reported. Use of specific antidotes or reversal agents has not been reported so far. To the best of our knowledge, there has not been any reported case of RSH associated with edoxaban or dabigatran. Neither was there any comparative studies regarding the clinical features of DOAC-associated RSH with those of non-DOAC-associated RSH. Interestingly, study on anti-factor Xa levels has not been reported in these case reports either (Table 1). Normal PT or international normalized ratio (INR) was documented in a few of them and it is well known that a normal PT or aPTT cannot rule out the therapeutic effect of DOAC [4]. Similarly, anti-factor Xa level was not determined in our patient as the diagnosis was not suspected from the start and the test was not readily available in our institution. Although PT and aPTT are reported to be insensitive to therapeutic concentration of apixaban [14], an elevated PT level which gradually normalized after drug withdrawal likely represented the effect of apixaban in our patient.

**Table 1:** Summary of published case reports on direct-acting oral anticoagulant-associated rectus sheath hematoma

Reference	Year	Country	Gender	Age	Symptoms	Underlying diseases	Type of DOAC	Clinical course
Kocayigit et al. [9]	2014	Turkey	F	75	Abdominal pain, dyspnoea and leg swelling	DM, HT, AF, MR, TR, pulmonary hypertension, Alzheimer’s disease	Rivaroxaban 10 mg daily	CT showed 5.5 cm RSH Hb 5.5 g/dL on admission INR 1.48; PT/aPTT/anti-FXa not reported Managed conservatively with FFP and PC transfusion Developed respiratory failure and died on 15^th^ days due to renal dysfunction and sepsis
Aktas et al. [10]	2016	Turkey	F	71	Abdominal pain and dyspnoea for 24 h Cough for 1 week due to URI	AF, CAD	Apixaban 5 mg bd	USG showed 11 × 7.5x3cm left RSH Hb 11.5 mg/dL and eGFR 33 ml/min/1.73m2 on admission Clotting profiles/anti-FXa not reported Managed conservatively and discharged 1 week later with apixaban 2.5 mg bd
Talari et al. [11]	2016	US	M	65	Right sided abdominal pain for 1 week; cough for several weeks after starting losartan	Metabolic syndrome, DVT of lower limbs and PE	Rivaroxaban (dose not specified)	CT showed 14.5 × 9x4.5 cm right RSH Hb dropped from 13.3 to 9.5 g/dL PT/INR were ‘normal’; aPTT/anti-FXa not reported Conservatively managed with pain control and PC transfusion No mention if anticoagulation was resumed
Gunasekaran et al. [12]	2017	US	F	68	Acute onset of severe abdominal pain in mid-epigastric radiating to back	DM, HT, hyperlipidemia, OSA, OA of knees, depression, left total knee replacement 1 month before admission, left leg DVT	Apixaban 10 mg bd	CT showed 10 × 4x17 cm left RSH Hb dropped from 9.7 to 7 g/dL ‘Normal’ PT/INR; aPTT/anti-FXa not reported Conservatively managed with PC transfusion IVC filter was inserted CT 1 month later showed resolution of RSH and aspirin 81 mg daily was added afterwards
Elango et al. [13]	2018	UK	F	69	Acute severe lower abdominal pain after severe coughing	AF	Apixaban (dose not specified)	CT showed 7.5 cm left RSH Hb dropped from 15.2 to 12.9 g/dL Clotting profiles/anti-FXa not reported Conservatively managed and apixaban withheld for 1 week Plan for resumption of apixaban by primary care physician
Borekci [5]	2019	Turkey	F	76	Acute abdominal pain after cough	DM, HT, AF, asthma, Hyperlipidemia, Hypothyroidism	Rivaroxaban 15 mg daily	CT showed 10 × 4x12cm left Hb 9.5 g/dL ‘Normal’ INR; PT/aPTT/anti-FXa not reported RSH with bleeding in retroperitoneum Conservatively managed with PC transfusion Discharged 7 days later with dabigatran 110 mg bd

*Anti-FXa*:Anti-factor Xa level, *aPTT* Activated partial thromboplastin time, *AF* Atrial fiberirllation, *bd* Twice per day, *CAD* Coronary artery disease, *CT* Computed tomography, *DM* Diabetes mellitus, *DVT* Deep vein thrombosis, *FFP* Fresh frozen plasma, *Hb* Hemoglobin, *HT* Hypertension, *INR* International normalized ratio, *IVC* Inferior vena cava, *MR* Mitral regurgitation, *OA* Osteoarthritis, *OSA* Obstructive sleep apnoea, *PC* Packed cells, *PE* Pulmonary embolism, *PT* Prothrombin time, *TR* Tricuspid regurgitation, *USG* Ultrasonography

The commonest symptoms and signs of RSH are acute abdominal pain, abdominal mass, tenderness and guarding, which could be indistinguishable from other surgical causes of acute abdomen. Tachycardia, diaphoresis, restlessness, hypotension and confusion are signs of hypovolemia which require urgent medical attention but are non-specific. Fothergill’s sign, where a mass remains palpable and does not cross the midline when the patient contracts his rectus muscle, and Carnett sign, where contraction of rectus muscle by sitting halfway up in a supine position exacerbates pain, are positive in RSH and may help to differentiate it from other intraabdominal pathologies. Abdominal ecchymosis, such as Cullen’s sign and Grey-Turner sign, indicates intraperitoneal rupture of RSH and is a late sign [2, 3, 6, 15, 16]. Therefore, an accurate diagnosis requires both a high index of clinical suspicion, coupled with appropriate radiological investigations. While ultrasonography of the abdomen is readily accessible and inexpensive with a sensitivity of 80–90%, CT scan is considered the gold standard for diagnosing RSH with 100% sensitivity and specificity [2]. CT also prevents unnecessary surgical interventions by excluding other intra-abdominal pathologies, provides information on the origin and extension of hematoma and helps in planning therapeutic strategy by classifying RSH by their anatomy [6].

Based on the radiographic features on CT scan, three types of RSH have been described [17]. Type 1 RSH is confined within the rectus muscle which does not dissect fascial planes or cross the midline and is almost always hemodynamically insignificant. Type 2 RSH is also confined within the rectus muscle but can dissect along the transversalis fascial plane or cross the midline. Type 3 RSH is usually large with extension below the arcuate line and there is often evidence of hemoperitoneum or blood within the prevesical space of Retzius as in our patient. While type 1 RSH can be managed conservatively (such as analgesic, compression of hematoma, packed red blood cell transfusion, fluid resuscitation, and replacement of clotting factors, for example, fresh frozen plasma, prothrombin complex concentrate and cryoprecipitate), surgical or radiological intervention may be required in type 2 and type 3 RSH [2]. Factors predicting failure of conservative treatment included active extravasation on CT angiography, larger hematoma volume and transfusion of ≥ 4 units of packed cells [18]. Evidence of ongoing bleeding (e.g., hemodynamic instability and active extravasation on CT), infection, bleeding into peritoneum, refractory pain and abdominal compartment syndrome represent potential indications for intervention. [2, 11, 19]. A recently published algorithm proposes that patients with hemadynamic instability, rapid blood loss (Hb drop ≥ 0.25 g/dL/hr and/or blood transfusion ≥ 3 units) and a hematoma size more than 7 cm are potential candidates for procedural intervention. Intervational radiology embolization, for example, microcoil, n-butyl cyanoacrylate (NBCA) and Squidperi embolization, is suggested to be the first treatment option after failed conservative management. Surgical intervention is reserved as a second-line treatment when embolization is unable to control the bleeding. Alternatively, surgery can be a first-line treatment for abdominal compartment syndrome. The relevant surgical procedures may include emergency ligation of the bleeding vessels (usually the inferior epigastric artery), laparotomy and clot evaculation [2–4, 20]. In two large retrospective series involving 156 and 126 subjects with RSH, 8 and 20% of them required some forms of intervention during the course of hospitalization respectively [2, 19].

Classically regarded as a benign condition with a favorable outcome, RSH is associated with an estimated mortality of 1.6–5% [2, 3, 6, 19]. Mortality occurs more commonly in patients with type 3 RSH, older persons, chronic kidney disease and patients on anti-coagulation [1–4, 7, 16, 19]. Resumption of anticoagulation after an index episode of spontaneous RSH is controversial and no guidelines or prospective studies exist to direct the appropriateness or timing of this issue. In a retrospective series by Cherry et al., 32.5% (*n* = 41) patients had anticoagulation reinitiated after the initial RSH and two developed recurrent RSH [2]. In another retrospective series by Kunkala et al. where 62% (*n* = 97) patients had their anticoagulation restarted during their hospitalization (median time 4 days after RSH), rebleeding occurred in 2 patients while thrombotic complications occurred in 5 patients due to interruption of anticoagulant therapy [19]. Evidence on anticoagulation resumption related to DOAC-associated RSH was even more limited with successful reinitiation reported in a few case reports only. [5, 10] Therefore, the decision to resume anticoagulation should weigh the risk of thromboembolism against the risk of rebleeding in future once the hematoma is considered stable. [13, 18, 21]

## Conclusion

RSH represents a potentially serious hemorrhagic complication associated with the use of DOAC. The spectrum of clinical features of this condition is yet to be fully elucidated given the scarcity of existing literature but acute onset of severe abdominal pain with significant drop in hemoglobin with or without hemodynamic compromise should alert clinician for such possibility. A high index of suspicion is required to direct appropriate investigation and early recognition. In addition to interruption of anticoagulation, supportive management with fluid resuscitation, packed cell transfusion and replacement of clotting factors forms the mainstay of treatment. The use of specific antidotes or reversal agents has not been reported and warrants future study. Resumption of anticoagulation after an index episode of RSH should balance the future risk and benefit of both bleeding and thromboembolism.

## Data Availability

Not applicable.
